# The missing voice of engagement: an exploratory study from the perspectives of case-managers at an early intervention service for first-episode psychosis

**DOI:** 10.1186/s12888-019-2315-0

**Published:** 2019-10-24

**Authors:** Rachel M. Tindall, Kelly Allott, Magenta Simmons, Winsome Roberts, Bridget E. Hamilton

**Affiliations:** 1 Orygen, The National Centre of Excellence in Youth Mental Health, Locked Bag 10, Parkville, Melbourne, VIC 3052 Australia; 2 Centre for Youth Mental Health, The University of Melbourne, Melbourne, Australia; 3 Department of Nursing, The University of Melbourne, Melbourne, Australia; 4 Department of Social Work, The University of Melbourne, Melbourne, Australia

**Keywords:** First episode psychosis, Qualitative research, Service engagement, Case-management

## Abstract

**Background:**

A key component of case-management in early intervention services for first-episode psychosis is engaging a person with the service and building a relationship from which therapy and treatment can be facilitated. The aim of this study was to understand how case-managers at an early intervention service experience the process of engagement and working with varying levels of attendance and participation.

**Methods:**

Qualitative interviews were conducted with the case-managers of nine young people treated at an early intervention service for first-episode psychosis within 6 months of treatment entry. Interviews discussed the process of working with the young person and factors that influenced service engagement. Interviews were analyzed using thematic analysis.

**Results:**

Case-managers described a range of influences on engagement which were grouped under the themes: young person and caregiver influences on engagement, case-manager influences on engagement, and influences of the early intervention service system on engagement. The experience of engagement was described as relational, however it occurred in the context of broader influences, some of which were unable to be changed or challenged by the case-manager (e.g., resource allocation, models of treatment, young person demographics).

**Conclusion:**

This study illustrates the challenges that case-managers face when working with young people with first-episode psychosis, and the direct influence this has on engagement with treatment. Understanding these challenges and addressing them in policy and service design may lead to improvements in young peoples’ recovery from first-episode psychosis and increase case-manager job satisfaction.

## Background

Developing services that provide comprehensive and timely treatment for first-episode psychosis (FEP) remains critical [1]. Early intervention services (EISs) provide flexible bio-psycho-social treatments which have been specifically developed to support recovery from FEP. In Australia, case-managers (nurses, allied health professionals, psychologists) and doctors provide this treatment over the first two to 5 years following initial presentation [2]. Treatment most commonly includes medications, community and social support linkages, support adjusting to symptoms and illness, cognitive behavioral therapy for psychosis and caregiver support. Levels of treatment assertiveness depend on service models, but the aim of EISs is to actively promote engagement by providing a service that is acceptable to service users [3].

Engagement in treatment is key to it being effective. Engagement is a dynamic construct that is unique to each individual. With EIS attrition rates at approximately 30% [4], it is vital to reflect on how services can better meet the needs of this diverse population. It is also important to note that attrition rates do not capture those who attend appointments but do not participate fully in them, or those who re-engage after a period of disengagement. This has been a significant limitation of previous quantitative studies [5], therefore fully understanding engagement demands a qualitative lens to capture these complexities.

Qualitative studies have explored how young people and caregivers experience engagement with EISs, as summarized in two recent meta-syntheses [5, 6]. More specifically, qualitative research has asked young people and caregivers to reflect on their individual journeys of engaging with an EIS. The personal and unique therapeutic relationship between the individual, their caregiver/s and clinician/s has been identified as integral to the success of engagement. However, there is no qualitative research from the perspectives of case-managers analyzing their experience of building and maintaining engagement with young people recovering from first episode psychosis.

Given the multifaceted nature of engagement, having the case-manager voice represented in the literature is critical [7, 8]. The therapeutic relationship and engagement are relational constructs and factors such as case-manager characteristics and approaches are anecdotally known to impact on their quality [9]. The therapeutic relationship may also be affected by administrative and performance factors which detract from clinicians’ capacity to maintain an open therapeutic relationship with a person [10]. Without understanding the actual experiences of all involved, and building this evidence base, attempts to improve engagement will be uninformed.

This qualitative study therefore aimed to understand the experience of case-managers when engaging young people with FEP in the early stage (first 6 months) of treatment. Informed by previous qualitative studies, the study focused specifically on the relational aspects of engagement. Case-managers were asked to reflect on working with a specific young person, which similar to previous qualitative studies with young people and caregivers, facilitated discussions around their experience of a distinct therapeutic relationship and the specific factors that affected the engagement or disengagement of that young person.

## Methods

### Study context

Nine young people, their caregiver (if applicable) and their associated case-manager were enrolled into a longitudinal qualitative study which aimed to understand experiences of engaging with an EIS from entry into the EIS, to 3 months post discharge. The EIS provides treatment for young people aged 15–25 years of age, for up to 2 years of consecutive treatment in a metropolitan area. This treatment may be provided at a clinic or at the person’s home (or other preferred place) depending on the needs of the young person. Cognitive-behavioural case-management is offered by the case-manager, alongside access to a psychosocial program, crisis team and inpatient care as needed. A case-manager working full time (40 h per week) works with 20–25 young people who are at varying stages of recovery from their first-episode psychosis. At the time of recruitment into the study, approximately 250 young people were receiving treatment in the EIS across two different clinic sites. At each site, approximately 15 case-managers worked either part-time or full-time. Case-managers in the EIS are required to have a clinical background (clinical psychology, social work, occupational therapy or nursing), with the majority of the EIS workforce consisting of clinical psychologists during the recruitment period.

### Sampling

Young person enrolment into the study attempted to replicate a real-world sample of young people in the EIS, with participant recruitment explained in detail in our paper exploring young person and caregivers experiences of entry into an EIS [11]. In summary, 45 young people who experienced a FEP and were referred into an EIS in Melbourne between July 2016 and March 2017 were approached to participate in the study. Inclusion criteria were broad to capture all young people who were referred into the EIS, with the only exclusion criterion being young people who had a clinical or personal relationship with a member of the research team. Young people were introduced to the study at, or before meeting their case-manager on initial entry into the EIS. The first nine young people who were consented into the study were followed at regular intervals through their journey with the EIS, along with their caregiver/s and case-manager/s.

This paper captures the views of the case-managers after a three- to six-month period of working alongside the young person, as there is a significant parcity of engagement literature from the perspective of case-managers during this critical time. Ethical approval was given by the local human research ethics committee [HREC/16/MH/131]. All participants provided written informed consent. Consent was also obtained from a parent or guardian for participants under 18 years old.

### Data collection procedures

Case-managers were approached and given a study overview when the young person had been with the EIS for between three and 6 months. During informed consent, participants were told that the information they disclosed would not be shared with the young person or caregiver in the study and identifying details would be removed prior to data analysis and dissemination.

Interviews (*N* = 9) were conducted at the EIS by the lead researcher. The interview schedule was developed with open-ended questions which aimed to encourage the participant to reflect on their personal experience of engagement; their role in the process of engagement; and any intrinsic or extrinsic factors that may precipitate young person disengagement from an EIS. Questions were guided by the findings of the research teams previous research in this area [5, 12]. The questions asked were:

1. Please tell me about young person’s current engagement with EIS
2. Please tell me what your engagement with young person in sessions is like
3. Please tell me about your therapeutic relationship with young person
  - Examples of good encounters
  - Examples of not so good encounters
  - What is your role?
4. What do you think could be influencing young person’s decisions to engage in treatment at EIS?
5. Can you tell me what you think young person’s treatment needs are?
  - What do you think their priorities for treatment are?
6. What support/resources do you draw on or need in ensuring you achieve engagement with young person?
  - Are these available to you?
7. When do you think discharge will occur?
  - What will need to happen before then?
8. Is there anything else you would like to say about working with young person?

One case-manager had two young people in the study and was interviewed separately about each young person. All interviews were audio-recorded and transcribed verbatim. Reflective field notes were written and included in analysis. After each interview was analyzed, participants were given their interview transcript and a letter outlining themes that arose during the interview to facilitate member checking. The median duration of the interviews was 30 min (ranging from 24 to 43 min).

### Data analysis

Interviews were thematically analyzed using the method described by Braun and Clarke [13]. This six-step method enables the researcher to identify patterns of meaning across the data in an inductive manner by: familiarizing themselves with the data; generating initial codes; clustering initial codes into related ideas; reviewing and checking themes in relation to the entire data set; redefining and naming themes; and synthesizing the themes and producing a report. Each interview was listened to several times and transcribed verbatim. RT studied each transcript line by line and developed an initial coding list using the analytical software Dedoose, a secure web-based software that facilitates identification and synthesizing of themes (www.dedoose.com). A sample of interviews were listened to and coded by a second member of the research team to minimise the risk of preconceived biases impacting the researchers understanding of the data. Differences were minor, with the research team iteratively consulting the data to make any refinements to the list of codes. A minimal amount of material was discarded. RT then integrated the codes / data into potential themes, which were discussed and grouped to identify connections and overarching themes. Emphasis was given to themes that were consistent across the sample.

Participants were rich informants who reflected on the questions in depth. The majority of themes had emerged by the sixth interview. Data saturation was reached by nine interviews, as is common for qualitative research, especially within such a homogenous sample [14].

## Results

### Participant overview

Case-manager and young person characteristics are described in Table 1. One case-manager had two young people in the study and was interviewed twice. Case-manager experience ranged from < 1 to > 20 years. Disciplines were social worker (*n* = 2); occupational therapist (*n* = 3); and clinical psychologist (*n* = 3).

**Table 1:** Participant demographics

	Case-Manager Demographics	Young Person Demographics
Case-study 1	Female, Occupational Therapist 8 years since qualification 2 years in EIS	Unemployed male in a de facto relationship. Aged between 19 and 24 years old. Diagnosis of schizophrenia. Cannabis and amphetamine use.
Case-study 2	Female, Occupational Therapist 2 years since qualification 1 year in EIS	Single, male student. Aged between 15 and 18 years old. Diagnosis of possible psychosis, with language deficits and depression. No substance use.
Case-study 3	Male, Clinical Psychologist 20 years since qualification 16 years in EIS	Single, male student. Aged between 15 and 18 years old. Diagnosis of hypomania and psychosis. No substance use.
Case-study 4	Female, Occupational Therapist 2 years since qualification 1 year in EIS	Single, male student. Aged between 15 and 18 years old. Diagnosis of schizophrenia. Cannabis use.
Case-study 5	Female, Clinical Psychologist 5 years since qualification 5 years in EIS	Single, unemployed male. Aged between 19 and 24 years old. Diagnosis of depression, anxiety and psychosis NOS. No substance use.
Case-study 6	Female, Social Worker 20 years since qualification 1 year in EIS	Female student in a de facto relationship. Aged between 19 and 24 years old. Diagnosis of Bipolar Affective Disorder with psychotic features. No substance use.
Case-study 7	Male, Clinical Psychologist 8 years since qualification 8 years in EIS	Single, male student. Aged between 15 and 18 years old. Diagnosis of schizophreniform psychosis and autism spectrum disorder. Cannabis use.
Case-study 8	Female, Occupation Therapist 1 year since qualification < 1 year in EIS	Single, female student. Aged between 19 and 24 years old. Diagnosis of depression and psychosis NOS. No substance use.
Case-study 9	Female, Social Worker 8 years since qualification < 1 year in EI	Single, unemployed female. Aged between 19 and 24 years old. Diagnosis of depression and psychosis NOS. No substance use.

The young people discussed were six males and three females, aged 15–24 years. Their overall demographics were consistent with the cohort of young people registered into the EIS over the recruitment period. Four young people identified as Australian, three identified as Australian and an additional nationality (English, Irish, Indonesian), one identified as Salvadoran and one identified as Indian. Seven young people were single throughout the study, one young person remained in a de facto relationship throughout the study, and one young person experienced a breakdown in his relationship during the study. Six young people were students and three young people were unemployed. Two young people had a forensic history and four young people had experience of using illicit substances.

Three overarching themes were identified from the case-manager participants (with six subordinate themes): (1) young person and caregiver influences on engagement (levels of engagement); (2) case-manager influences on engagement (building trust and connection; relational approaches to engagement; strategies to promote engagement; and individual case-manager qualities) and (3) influences of the EIS system on engagement (the nuances of time).

### Young person and caregiver influences on engagement

#### Levels of engagement

The EIS model of care promoted assertive engagement with young people, particularly in the initial months following a FEP. Alongside this, symptomatic and functional recovery from the FEP was anticipated for all young people attending the EIS. This was observed throughout the interviews, with all case-managers actively attempting to engage the young people they worked with and holding hope for the young person’s recovery. Disengagement was perceived as episodic and something that could be altered, rather than an absolute.

Case-managers spoke about young people who were actively (*n* = 6) and passively (*n* = 3) engaged with the EIS. Six young people were actively eager to have therapy to gain an understanding of their experiences. They attended the majority of appointments and would only miss appointments if unwell or if there were competing priorities. This active engagement was related to case-managers feeling a sense of momentum in the young persons’ recovery and enjoyment from working together.

The three young people who were passively engaged attended appointments if they were brought in or seen at home but did not initiate contact or independently attend appointments. Whilst appreciating help, there was a sense of apathy around their role in engagement. This was associated with frustration and disappointment for the case-manager, who wanted to actively engage them and support their recovery from the FEP:

*“I went last week to go get him [young person] and he wasn't there, and I waited for maybe 15 minutes, knocking on the door, thinking, okay he might be asleep. And after 15 minutes, there was nothing, so I left … So, I think frustration is a big part of it.”* (Case-manager 4)

Case-managers reflected on why disengagement was occurring and their own role in the process:

*“Sometimes it's hard to not personalize non-attendances or periods of disengagement and feel a bit like, is there something that I'm not bringing, or am I not giving her what she needs or- and that can be sort of a hard thing to navigate as a case manager with any young person.”* (Case-manager 9)

Differences in engagement patterns meant treatment was both delivered in different venues (home versus clinic appointments) and involved different amounts of content. Desire and enthusiasm to engage were not the only factors that impacted this, but also practical (e.g., financial, transport), psychological (e.g., residual symptoms, side-effects from medications) and support (caregiver involvement and capacity to actively provide support) factors.

### Case-manager influences on engagement

#### Building trust and connection

‘Case-management’ was described under four domains: understanding the young person (e.g., building a formulation); mental health treatment (e.g., making sense of experiences); psychosocial support (e.g., supporting peer connections); and caregiver support. To facilitate all aspects of treatment, connection and trust needed to be established and maintained between case-manager, young person and if applicable, caregiver.

Investing time and maintaining consistency within the treating team were themes that were prevalent when discussing building trust and connection. An example of this came from a case-manager discussing her relationship with a young person who attended few appointments:

*“It's challenging in terms of engagement and access and being able to have that kind of continuity and so I don't really feel like we've achieved necessarily a whole lot in the time that we have worked together but I suppose the fact he's saying he doesn't want someone new and he does come in when he can … There’s definitely potential to continue working there.”* (Case-manager 1)

Without consistency, it was felt that the young person would not have maintained engagement with the service.

The other important aspect of building trust and connection was the importance of investing time in a young person even if not present for appointments, by maintaining contact by phone, text messages or outreach. This helped build trust over time and offered alternative opportunities for connection.

#### Relational approaches to engagement

Case-managers used a range of relational approaches to enhance engagement, including showing an interest in the young person and maintaining a collaborative approach. It was evident that case-managers had individualized approaches to each young person, their core aim being to understand the young person’s experiences and make themselves useful to the young person:

*“We talked about [his interests] and I guess I made a bit of a pitch about what we could do for him, in engaging with [EIS] as well. And actually, it's a bit ridiculous but I remember leaving him and thinking, ‘I think this will go alright’ cause he was like, ‘You're a cool dude!’ So, it must have been a reasonably okay first experience for him.”* (Case-manager 7)

Working collaboratively with the young person afforded opportunities for trust and connection to build, and empowered the young person to drive the focus and pace of their treatment:

*“I guess for me as a clinician I do focus very strongly on rapport building and taking cues from the young person, so not sort of rushing or pushing anything so hopefully that's created a sense of [young person] feeling quite comfortable and maybe like she's in charge of the pace that we progress.”* (Case-manager 9)

However, balancing collaboration when there was passive engagement was difficult:

*“I think we obviously have some goals in mind. I'm always mindful in having done recovery plans and stuff that you have your own stuff which is really about keeping people well … but I think it needs to be driven outside of that. It needs to be driven by the client … Which is not always easy if they don't want to be here or, you know, if they don't actually have any particular goals”.* (Case-manager 6)

### Strategies to promote engagement

Case-managers believed that a number of approaches and strategies promoted engagement. For example, one case-manager described the value of conversations that occurred in a less formal setting, for example in the car:

*“And also, the fact that when you are sitting in an office, kind of face to face, there's a different interaction between sitting in a car, listening to music. He knows that I have my iPod every time and he knows that I've got a cord and he can plug his phone in, so the music really helps with the engagement as well. And he's shown me some of his favorite rappers, and I've shown him some of mine, that wouldn't happen in an office.”* (Case-manager 4)

Case-managers also described strategies used during sessions to promote a therapeutic space. These included balancing the involvement of others (caregivers, treating team, other services) and considering the use of language to be fully understood by the young person. When engagement was difficult, case-managers sought advice and support from the broader EIS team through weekly clinical reviews, clinical supervision or informal contacts. However, engagement was predominantly described as individual interactions within the young person – case-manager relationship, and as such, there was a sense of case-managers working in isolation from the broader team.

#### Individual case-manager qualities

The way case-managers worked with the young person was also influenced by specific factors that they brought to the relationship. An example of this was their level of experience and the way clinical risk was addressed within the context of treatment, recovery and the therapeutic relationship:

“*There’s specific topics that I feel like we kind of, she's talking in one direction and I might be struggling to follow. So especially around like suicide and self-harm. I find these topics really challenging to kind of make her feel safe and elicit the information that I'm trying to find out*.” (Case-manager 8).

Different disciplines also approached recovery goals in different ways, for example, occupational therapists tended to focus on meaningful activities, and psychologists tended to focus on therapy. The ability to do discipline specific work influenced job satisfaction:

*“He’s a really good client to have, l really enjoy catching up with [young person] and as far as my caseload goes, [young person] is a breath of fresh air. He’s a good therapy client, he engages really well, and I enjoy his company.”* (Case-manager 5)

Case-manager gender or age was noted by two case-managers, but not identified as a significant advantage or deterrent to engagement. Instead, the relational approaches and a commitment to investing time in the young person were identified as more important facilitators of engagement.

### Influences of the EIS system on engagement

#### The nuances of time

Time was discussed in positive and negative terms. Entering into treatment with the knowledge that this could be offered over a period of 24 months supported engagement:

*“We’ve got plenty of time to work with him, so that immediately took the pressure off both of us, which I think, paradoxically, actually meant that he was able to be more open, because he didn't feel under any kind of pressure to do that.”* (Case-manager 3)

Appreciation of time was echoed by all case-managers, who communicated to the young person and their caregivers that there was someone there for them longer-term.

However, there was also a perceived lack of time to do all that was needed to provide comprehensive and holistic care. This was attributed to the quantity and acuity of young people on a case-managers caseload, staff turnover and administrative tasks. Case-managers worried about the impact of this on their capacity to build trust and connection with the young person, and to follow through on priorities. There was also concern that when disengagement occurred, this impacted the overall time and treatment that young people had available to them, as time with the service was two consecutive years with no available options to advocate for additional time. Case-managers described a balance between desired care provision and actual care provision:

*“I do feel like [young person]’s someone, if you know there was a period of disengagement, and I said to her, “Hey, let me meet you at your local cafe and shout you lunch and we'll catch up then”:, that probably would work really well, but there's just not time or money to do that kind of work really, it is quite difficult.”* (Case-manager 9)

Lack of time also impacted on the case-managers’ capacity to engage in activities such as clinical supervision, session planning and reflection. Case-managers felt that not having this space available could have led them to only be partially present in sessions at time, and this may have impacted on the quality of their therapeutic relationship with the young person:

*“There are times when I might have a session and maybe, you know, I think often it's about where I’m at and where my head's at … nothing really kind of comes out of it, or I feel like nothing's really come out of it. I don't know necessarily how she would feel.”* (Case-manager 6).

Young person engagement and associated work satisfaction, which may impact staff turnover and available clinical resources, was therefore both impacted by, and contributed to, service level factors.

## Discussion

**Fig. 1:**
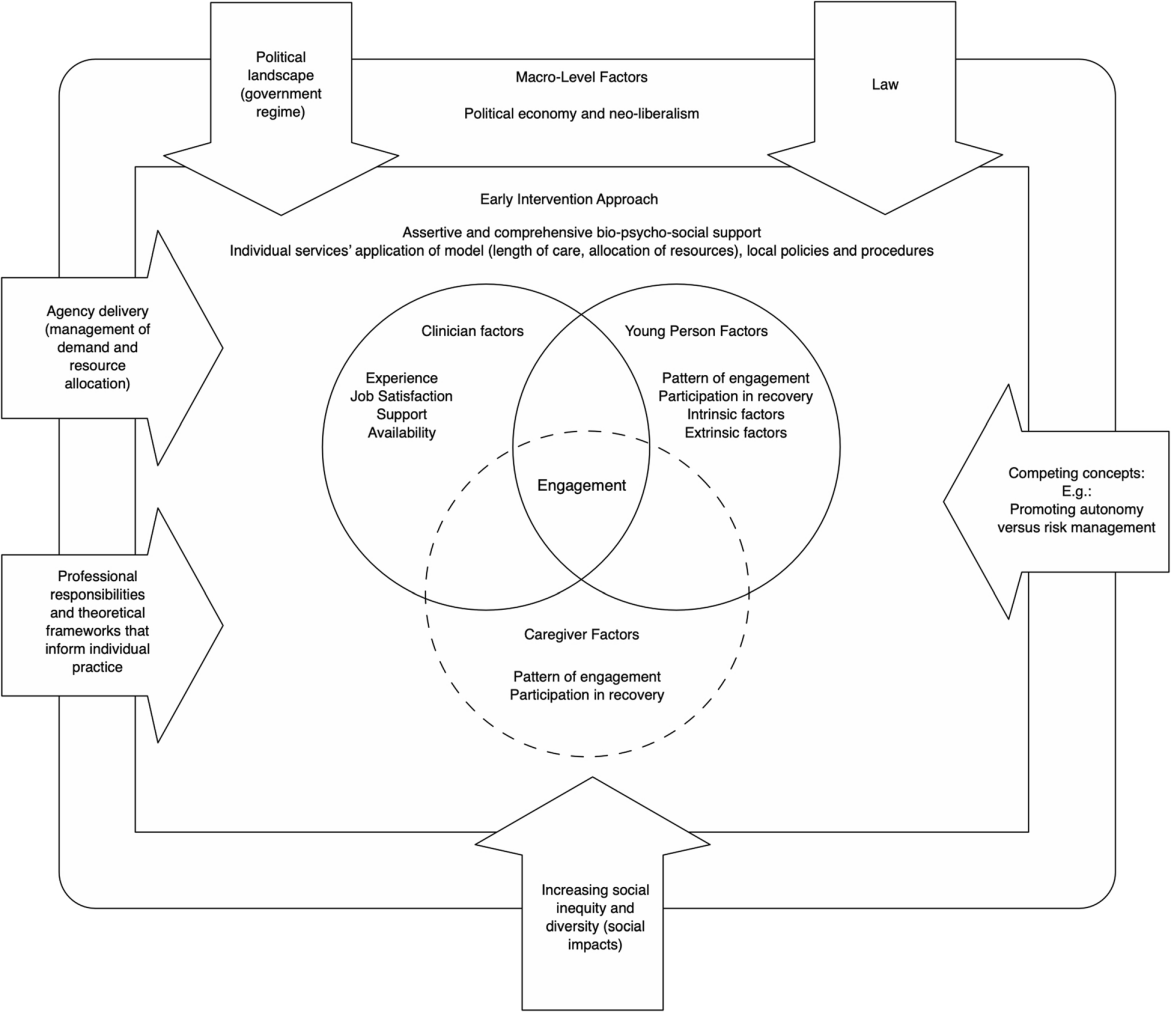
The operational and human constructs of engagement

Case-managers spoke of engagement as a multifaceted process that they endeavoured to facilitate. Many factors were outside of their control (e.g., young person and caregiver factors; influences of the EIS system). Despite this, they held responsibility for engaging the young person in treatment. This led to a strong focus on factors that they brought to the engagement process (case-manager influences). They worked within the resources available to them to creatively connect with the young person and build trust. The complex environment in which this engagement occurred is shown in Fig. 1. To our knowledge, this is the first study to discuss what is anecdotally known about the limitations of engaging young people with FEP within current EIS service systems, but not evidenced within the existing literature.

Case-managers described the young people in the study as having varying levels of participation and engagement. This builds upon previous studies that have conceptually explored treatment participation [15] and studies that have described engagement as a dynamic process that occurs on a spectrum [12]. It is interesting that given attempts to recruit a real-world sample of young people, most (six) participants were described as experiencing good engagement. This may be due to a research participant bias, i.e., people who consent to research may be those who are more engaged with a service. Or this may highlight the inherent need for support experienced by young people after experiencing a first episode psychosis, especially if they engage with the EIS in some capacity (i.e., not those who do not engage with the EIS at all).

Previous research has also suggested that individual circumstances and motivations create a need that aligns with services and treatments, and this directly influences the level of service engagement [16]. From this study, all young people had a need that the EIS could meet. When a case-manager was available to a young person who was passively engaged, for example through outreach, the young person interacted with the case-manager, and there was engagement within that interaction. Often the person’s need was to understand the loss of identity experienced with psychosis, and to rebuild autonomy [11]. This contrasts with previous findings in studies with adult cohorts, for example Priebe, Watts, Chase and Matanov [17] who found that loss of identity and autonomy fueled disengagement with mental health services.

Reasons for passive engagement were commonly related to practical (e.g., financial, transport), psychological (e.g., residual symptoms, side-effects from medications) and support (caregiver involvement and capacity to actively provided support) factors. This is of particular interest as structural adversity, trauma and poor community support are some of the key reasons for poor treatment response and outcomes in this population [18]. In a society with increasing inequity and diversity, ensuring that flexible and assertive services can be offered to these individuals is of upmost importance. Intensive case-management models, which include resourcing for outreach and more assertive treatment, should therefore be considered as a service priority for this population [19].

The qualities of the individual case-manager also contributed significantly to the engagement process. Case-managers brought both static (e.g., experience, gender, profession) and dynamic (e.g., motivation, fatigue) characteristics to each relationship. Static demographic factors were not identified to be a primary deterrent or motivator for engagement, with personal characteristics, such as a collaborative and relaxed approach, being of more value. This correlates with what young people and their caregivers identified in Tindall, Allott, Simmons, Roberts and Hamilton [11]. However, case-managers identified having greater job satisfaction when the goals of the therapeutic relationship were matched with their specific professional skills (e.g., occupational therapist enjoying a focus on building meaningful activities). Given the generic role of most case-management roles in EISs, and the increasing administrative tasks associated with the roles, the concept of matching young person and case-manager depending on needs/skills may have better outcomes [10].

The dynamic risk factors of motivation and fatigue described by some case-managers have the potential to influence any contact. As poor relationships with case-managers are a significant contributor to disengagement [20], the risks associated with these factors are high. Some of these risks may be mitigated through support, supervision and the team approach taken by the EIS. However, as engagement was described to occur within the therapeutic relationship and not within a broader team environment, the importance of these team supports may not be fully realised.

The EIS model of assertive engagement appeared to be adopted by all case-managers interviewed and was central to their reflections on each young person’s level of engagement. Case-managers also spoke optimistically of the potential for both functional and symptomatic recovery for each of the nine young people in the study. It is likely that this sense of hope and the onus on the case-manager to facilitate engagement translated through to their contacts with the young person and their caregivers, protecting against disengagement.

Case-managers placed importance on being collaborative in their approach with young people. Similar findings were identified from the analysis of young person and caregiver data from this study [11]. This aligns with the recovery movement, which promotes the importance of clients taking risks and clinicians tolerating the possibility of failure [21]. However, given that case-managers also felt responsible for building and maintaining engagement, there lies a tension between assertively demonstrating care, and supporting young person’s choice. O’Keeffe, Sheridan, Kelly, Doyle, Madigan, Lawlor and Clarke [22] add further context to this tension by examining client perspectives of legislative shifts towards recovery focused care. In a cohort of people who were recruited from an EIS for FEP and followed up over 10 years, experiences of recovery orientated care for those who did not achieve symptomatic and functional recovery were associated with feelings of abandonment by services. Assertive engagement is an integral aspect of the EIS service model [3]. How individual services manage and deliver this model varies across sites; however, at the EIS studied, these principles were apparent and valuable.

Alongside the young person and case-manager factors sits the greater macro-level factors impacting mental health service delivery. These range from law (e.g., mental health acts; management of clinical risk) to the broader political landscape (e.g., resource allocation). This became apparent in this study in relation to time as a resource. It was found that good engagement is associated with a longer treatment period, but it remains difficult to meet the individual needs of each client, given caseload sizes and other demands. Given the potential negative impact of this, advocacy for the resources to provide assertive approaches to engagement and a substantial treatment time should continue at both an individual service level and within the larger political environment.

The experiences described by the case-managers in this study are shared amongst health professionals from broader mental health treatment services. Internationally, many countries have established Assertive Outreach Teams (AOTs) to enhance treatment provision for clients with serious mental illnesses who are difficult to engage, have high levels of need and experience frequent psychiatric inpatient admissions [23]. These teams are similar in structure to EISs, as they aim to have small caseloads and can therefore be more flexible in their treatment approaches. It is interesting, but not surprising to realise that the concepts of time (longer treatment periods to invest in the person) and the therapeutic relationship are themes that have emerged from the qualitative engagement literature conducted with clinicians in AOTs [17, 24, 25]. The perceived paucity of time in generic community mental health services is also highlighted by consumers who have experienced both an AOT and a community mental health team [26]. If there is to be any change in the overall experience of engaging with mental health services, and consequently mental health outcomes, all services could benefit from adapting components of AOT and / or EIS models of care.

Although this study provides valuable insights into the case manager experience of engagement, there were limitations. The researcher was associated with the EIS and despite careful consideration to dual relationships and confidentiality, this may have impacted on participants willingness to openly reflect. The findings of this study may also be site specific due to large variabilities in the way EISs are resourced and delivered. Interestingly, there were no case-managers from a nursing background involved in the study at this data collection point, which limits the understanding and comparability of the nursing experience of engaging young people with FEP in EISs.

## Conclusions

The importance of actively offering care to young people after a FEP cannot be overstated. EISs must consider the demographics of their young people, offering a service that is flexible to their needs. This allows the opportunity for all young people to be offered the opportunity to engage, regardless of their socioeconomic background, illness or support systems. Resources to facilitate this include time, capacity to outreach and opportunity for alternative modes of engagement. Whilst this is built into the theoretical ideal of EISs internationally, the reality often varies due to resources and diverse applications of the model. Alongside this, considering and matching individual case-manager skills to young person needs may also enhance young person outcomes and case-manager work satisfaction.

## Data Availability

The datasets used and analyzed during the current study are not publicly available due to the sensitive and personal information disclosed during the qualitative interviews. De-identified data are available from the corresponding author on reasonable request.

## Abbreviations

AOT: Assertive Outreach Teams
EIS: Early Intervention Service
FEP: First Episode Psychosis
